# Increased hand washing reduces influenza virus surface contamination in Bangkok households, 2009–2010

**DOI:** 10.1111/irv.12204

**Published:** 2013-11-07

**Authors:** Jens W Levy, Piyarat Suntarattiwong, James M Simmerman, Richard G Jarman, Kara Johnson, Sonja J Olsen, Tawee Chotpitayasunondh

**Affiliations:** a International Emerging Infections Program, Thailand MOPH- US CDC CollaborationNonthaburi, Thailand; b Queen Sirikit National Institute of Child HealthBangkok, Thailand; c Influenza Division, Centers for Disease Control and PreventionAtlanta, GA, USA; d U.S. Armed Forces Research Institute of Medical SciencesBangkok, Thailand; e Harvard Medical SchoolBoston, MA, USA

**Keywords:** Human, influenza, Thailand, transmission

## Abstract

Within a hand-washing clinical trial, we evaluated factors associated with fomite contamination in households with an influenza-infected child. Influenza virus RNA contamination was higher in households with low absolute humidity and in control households, suggesting that hand washing reduces surface contamination.

## Introduction

Understanding the mode by which influenza is transmitted is important for implementing effective control strategies. The importance of indirect (fomite) transmission, compared with direct (droplet) and aerosol transmission, remains uncertain. While studies have demonstrated that influenza viruses can survive in the environment,^1^,^2^ human transmission from fomites has never been documented.^3^ Recent evidence suggests that absolute humidity (AH) is more relevant to influenza transmission than relative humidity.^4^,^5^ In an earlier study performed in urban Thai households with a child with laboratory-confirmed influenza during the 2009 pandemic, we found that influenza virus surface contamination was significantly associated with lower age of the index case and seasonal (versus pandemic) influenza strains.^6^ We extended data collection in 2010 primarily to increase the sample size to evaluate the effect of hand washing and absolute humidity on the presence of influenza virus contamination on surfaces.^6^

## Materials and methods

Both the 2009 and the present studies were nested within a randomized controlled trial (RCT) to evaluate non-pharmaceutical interventions to prevent the transmission of influenza in families of a child with laboratory-confirmed influenza identified from a large public pediatric hospital serving the urban working class in Bangkok.^7^ We enrolled children 1–15 years of age with onset of influenza-like illness (ILI) within 48 hours previous to an outpatient clinic visit in a large Bangkok children's hospital. Households of children positive for influenza by rapid test (QuickVue Influenza A+B, Quidel) were enrolled and randomized to the study arms (control, hand washing, hand washing and face mask). Study nurses collected data and nasal and throat swabs from the index child and household members during home visits within 24 hours (day 0/1) and on days 3 and 7 of clinic visit. Frequency of hand washing of the index child was ascertained in the hand-washing arm by logs at the start of follow-up (the relevant exposure period for subsequent surface contamination and secondary infections) or retroactively in the control arm on day 7 by interview (so as not to contaminate the control arm). This study was approved by an institutional review board of the US Centers for Disease Control and Prevention (CDC) and the Queen Sirikit National Institute of Child Health, Thailand. Detailed methods may be found in the 2009 surface contamination substudy.^6^ Briefly, beginning in July of the study year, we approached every household enrolled in only the control and hand-washing households of the RCT to participate in the substudy until reaching our target enrollment (100 households in 2010). We swabbed a 1) bathroom door handle 2) refrigerator door pull 3) television remote control 4) light switch in main room 5) frequently used child's toy with a non-porous surface and 6) phone. We also swabbed the finger pads on the dominant hand of the index patient and any household members with ILI. We used handheld, calibrated psychrometers (Extech Instruments Model RH390; Flir Company) to measure the dew point which is a metric of AH.^6^ Swabs and AH measurements were taken on day 3 in the 2009 study and on days 0/1, 3 and 7 in 2010.

Real-time reverse transcription–polymerase chain reaction (rRT-PCR) was performed on individual swab specimens.^8^ rRT-PCR-positive specimens were evaluated for cytopathic effect on Madin–Darby canine kidney cells and when observed hemagglutinin assays were performed on the supernatants.^9^ We report results from the day 3 sampling from this study separately and combined with the first study. We used prevalence (of any surface contamination in a household) risk differences (PRDs) and 95% Wald confidence intervals as the measure of association. We categorized the continuous variables of age of the index case, AH and temperature based on the simple dichotomy above and below median values on day 3 in the corresponding data set (i.e. the second study period alone or combined with the first). We performed stratified analyses to examine the effect of the exposure on surface contamination across strata of one variable at a time. Effect measure modification was evaluated as departures from additivity.^10^ We considered a *P*-value of <0·10 for the test of homogeneity to suggest effect measure modification. Analysis was conducted using sas 9·2 (Cary, NC), except for stratified analyses conducted with Rothman's Episheet.^11^

## Results

### Current study (2010)

We enrolled 108 households during June 25 to November 12, 2010, of which 101 completed 7 days of follow-up (three control and four hand-washing households withdrew by day 7). The number of index children with positive finger swabs on days 1, 3 and 7 was 26 (26%), 23 (23%) and 3 (3%), respectively. The number of households with at least one positive surface on days 1, 3 and 7 was 9 (9%), 8 (8%) and 4 (4%), respectively. On day 3, the surface positivity was 12% in the control arm and 4% in the hand-washing arm (PRD 7·8%; 95% CI: −2·6 to 18·1; *P* = 0·15; Table 1). Households with lower dew points had significantly more surface positivity (15%) compared with those with higher dew points (2%; PRD 12·7%; 95%CI: 2·0–23·3%; *P* = 0·02). Surface positivity was 10·8% in the households with other secondary influenza infections and 6·3% in those without (PRD 4·6%; 95% CI: −7·1–16·2%; *P* = 0·41).

**Table 1:** Unadjusted prevalence risk differences for influenza RNA surface contamination, 2009–2010, Bangkok, Thailand

	Second study (2010) *n* = 101 households				Both studies (2009, 2010) *N* = 191 households			
		
	No. positive*/total	Prevalence risk difference			No. positive*/total	Prevalence risk difference		
				
	(%)	(%)	(95% CI)	*P*	(%)	(%)	(95% CI)	*P*
All	8/101 (7·9)				24/191 (12·6)			
Study arm								
Control	6/51 (11·8)	7·8	(−2·6,18·1)	0·1485	17/96 (17·7)	10·3	(1·1,19·6)	0·0310
Hand washing	2/50 (4·0)				7/95 (7·4)			
Gender of index patient								
Female	5/43 (11·6)	6·5	(−4·7,17·6)	0·2349	13/84 (15·5)	5·2	(−4·4,14·8)	0·2820
Male	3/58 (5·2)				11/107 (10·3)			
Age								
Less than or equal to median	5/61 (8·2)	0·7	(−10·0,11·4)	0·8991	14/96 (14·6)	4·1	(−5·3,13·4)	0·3977
Above median	3/40 (7·5)				10/95 (10·5)			
Influenza category								
Seasonal (H3N2, H1N1, B)	3/50 (6·0)	−3·8	(−14·3,6·7)	0·4791	11/76 (14·5)	3·2	(−6·6,13·0)	0·5178
A(H1N1)2009pdm	5/51 (9·8)				13/115 (11·35)			
Dew point in household								
Less than or equal to median	7/48 (14·6)	12·7	(2·0,23·3)	0·0197	18/93 (19·4)	13·2	(3·8,22·5)	0·0063
Above median	1/52 (1·9)				6/97 (6·2)			
Secondary influenza infections in household								
≥1 case	4/37 (10·8)	4·6	(−7·1,16·2)	0·4135	11/63 (17·5)	7·3	(−3·4,18·0)	0·1522
None	4/64 (6·3)				13/128 (10·2)			
Reported hand washing of index case (times/day)								
Less than or equal to median	6/66 (9·1)	2·8	(−8·0,13·7)	0·6300	19/113 (16·8)	9·6	(0·3,18·8)	0·0642
Above median	2/32 (6·3)				5/69 (7·3)			

* Positive for influenza RNA by rRT-PCR from ≥ 1 or 6 surfaces tested on day 3 after onset of symptoms.

### Comparison of current (2010) and prior (2009) study

Influenza B virus was more frequently identified among index cases in 2010 compared with 2009 [29/101 (28·7%) versus 1/90 (1·1)%; *P* < 0·0001], and the average age of the index case was younger in 2010 (5·3 versus 7·6 years; *P* ≤ 0·0001). During 2010, index patients reported washing their hands less frequently (2·9 times, SD = 1·9/day) compared with those in 2009 (3·8 times, SD = 2·4; *P* = 0·007). In 2010, households had higher dew points (24·3, SD = 1·6 versus 23·6, SD = 1·3; *P* < 0·0001) compared with those in 2009.

### Combined analysis (2009–2010)

There were 191 households (95 hand-washing and 96 control households). Reported hand washing of the index child was significantly higher in the hand-washing compared with control households (Table S1; 3·9 versus 2·8 episodes; *P* = 0·001). There were no differences between the intervention arms with respect to sex and age of the index child, or distribution of index patient infections between seasonal influenza viruses and influenza A(H1N1)pdm09 (Table S1) or time between fever onset and first home visit (data not shown). AH on day 3 was also similar between the intervention and control groups. The percent of households with ≥1 secondary infection on the day 3 home visit was similar in the hand-washing households (35, 36·8%) as control households (28, 29·2%; *P* = 0·26), suggesting similar viral shedding between the two study arms. Notably, the percent of households with secondary infections in the hand-washing and control arms on day 3 and 7 combined (39·0 versus 27·1%, *P* = 0·08) is in the opposite direction than expected if hand washing prevented infection.

Overall, 24 (12·6%) households had ≥1 rRT-PCR-positive surfaces on day 3 (three households had two and 21 had one). No live viruses were cultured from any surface. The TV remote control and plastic toy were the most frequent positive surfaces (nine households each) followed by the bathroom door knob (three households), a light switch in common area, refrigerator door handle and phone (two households each). In the 13 households in which the virus from the surface swab sample was subtypable, the strain matched that of the index patient.

Seventeen (17·7%) control households had a rRT-PCR-positive surface compared with 7 (7·4%) of hand-washing households (PRD 10·3%; 95%CI, 1·1–19·6%; *P* = 0·03; Table 1). The results based on actual reported hand use, although subject to bias due to differences in ascertaining hand-washing behavior, support the intent to treat analysis. A total of 16·8% of households with ≤ median index case, hand washing had surface positivity compared with 7·3% of households >median (PRD 9·6; 95%CI: −0·3–18·8%; *P* = 0·06). Households with ≥1 secondary influenza infection had a non-significant increase in household surface positivity compared with those without a secondary infection (PRD 7·3%; 95%CI: −3·4–18·0%; *P* = 0·15). Nineteen percent of households ≤ median dew-point value had a positive surface, compared with 6·2% of households above the median value (PRD 13·2; 95% CI: 3·8–18·8%; *P* ≤ 0·01).

Because only 24 households had a positive surface, we looked at the association between the two primary exposures (study arm and AH) and outcome (surface swab positivity) stratified by one variable at a time. The PRD between study arm and surface positivity was not confounded by any variables [adjusted PRDs (9·5–11·0%) similar to unadjusted PRD (10·3%)], but we did find evidence of effect measure modification (Table S2a). In the study arm exposure, the excess risk of surface contamination in the control households was significantly higher in households with a low AH (*P* for effect modification <0·01) and in households with secondary infections (*P* for effect modification <0·05; Table S2a). In the AH exposure, the excess risk of surface contamination in households with a low AH was significantly higher in households with secondary infections (*P* for effect modification =0·01; Table S2b).

## Discussion

Although about 25% children infected with an influenza virus in the 2010 study had a positive finger swab, surface swab positivity of household objects was low (<10%) and dropped over the course of a week. To evaluate the importance of hand washing to reduce this low-level positivity, we did a combined analysis of 2009 and 2010 study data.

The independent findings of increased surface contamination in control and low-humidity households suggest that hand washing and high humidity reduce the presence of virus on surfaces and so maybe relevant to fomite transmission. That these effects on surface contamination existed primarily in households with secondary infections is relevant as these households are likely to have more virus in the atmosphere for the effects to be apparent.

The correlation between low AH and higher prevalence of contamination supports earlier reports that AH is an important variable with respect to environmental persistence of influenza virus.^4^ Our findings also support current hand-washing guidance to reduce surface contamination and thus the potential for indirect contact transmission of influenza. However, in this study, no live viruses were cultured from the rRT-PCR-positive surface samples and we did not observe any relationship between hand-washing and secondary influenza infection in this nested study or the larger randomized control trial.^7^ Furthermore, a mathematical modeling study of the Bangkok RCT and a similar study in Hong Kong found aerosol transmission to be the dominant transmission route.^12^ Taken together, these findings suggest that in urban Bangkok households with an influenza-infected child, aerosol, droplet and direct contact transmission may be relatively more important than indirect modes of transmission via surface contamination. Future studies would benefit from larger sample sizes, more precise measurement of hand-washing and more varied household environmental conditions.

## Supporting Information

**Table S1.** Distribution of covariates by primary exposure and study arm (hand washing vs. control). **Table S2.** (a) Stratified analysis of hand washing versus control arm and surface contamination with influenza viruses, 2009–2010, Bangkok, Thailand. (b) Stratified analysis of absolute humidity (high vs. low dew point) and household surface contamination with influenza viruses, 2009–2010, Bangkok, Thailand.
