# Supplementary material for: Increased hand washing reduces influenza virus surface contamination in Bangkok households, 2009–2010
Source: Influenza Other Respir Viruses. 2013 Nov 7;8(1):13–6. doi: 10.1111/irv.12204 (PMC4177792; doi:10.1111/irv.12204)
Supplement: Supplementary file 1 [file irv0008-0013-SD1.doc]

Supplemental table 1. Distribution of covariates by primary exposure and study arm (hand washing vs. control).

|  | | | | | | | | | | |
| --- | --- | --- | --- | --- | --- | --- | --- | --- | --- | --- |
|  | Second Study | | | | |  | Both Studies | | | |
|  |  | |  |  |  |  |  |  |  |  |
|  | All | | Hand wash | Control | P |  | All | Hand wash | Control | P |
| All | 101 | | 50 (50) | 51 (50) |  |  | 191 (100) | 95 (50) | 96(50) |  |
|  |  | |  |  |  |  |  |  |  |  |
| Gender |  | |  |  |  |  |  |  |  |  |
| Male | 58 (57.4) | | 28 (56.0) | 30 (58.8) |  |  | 107 (56.0) | 51 (53.7) | 56 (58.3) |  |
| Female | 43 (42.6) | | 22 (44.0) | 21 (41.2) | 0.774 |  | 84 (44.0) | 44 (46.3) | 40 (41.7) | 0.5180 |
|  |  | |  |  |  |  |  |  |  |  |
| Age, mean (+/- SD) | 5.3 (+/- 3.6) | | 5.2 (+/-3.5) | 5.0 (+/- 3.7) | 0.705 |  | 6.4(+/-3.7) | 6.3 (+/-3.7) | 6.5 (+/-3.7) | 0.7590 |
| Above median | 40 (39.6) | | 20 (40.0) | 20 (39.2) |  |  | 95 (49.7) | 46 (48.4) | 49 (51.0) |  |
| Less than or equal to median | 61 (60.4) | | 30 (60.0) | 31 (60.8) | 0.936 |  | 96 (50.3) | 49 (51.6) | 47 (49.0) | 0.7172 |
|  |  | |  |  |  |  |  |  |  |  |
| Influenza category |  | |  |  |  |  |  |  |  |  |
| A(H1N1)2009pdm | 51 (50.5) | | 25 (50.0) | 26 (51.0) |  |  | 115( 60.2) | 55 (57.9) | 60 (62.5) |  |
| Seasonal (H1N1, H3N2 ,B) | 50 (49.5) | | 25 (50.0) | 25 (49.0) | 0.922 |  | 76 (39.8) | 40 (42.1) | 36 (37.5) | 0.5156 |
|  |  | |  |  |  |  |  |  |  |  |
| Dew point in household, mean (+/- SD) | 24.3 (+/- 1.6) | | 24.5 (+/-1.5) | 24.2 (+/-1.7) | 0.363 |  | 24.0 (+/-1.5) | 24.1 (+/-1.4) | 23.9 (+/-1.5) | 0.3584 |
| Above median | 52 (52.0) | | 28 (57.1) | 24 (47.1) |  |  | 97 (51.1) | 51 (54.3) | 46 (47.9) |  |
| Less than or equal to median | 48 (48.0) | | 21 (42.9) | 27 (52.9) | 0.313 |  | 93 (48.9) | 43 (45.7) | 50 (52.1) | 0.3822 |
|  |  | |  |  |  |  |  |  |  |  |
| Secondary influenza infections in household |  | |  |  |  |  |  |  |  |  |
| >1 | 64 (63.4) | | 29 (58.0) | 35 (68.6) |  |  | 128 (67.0) | 60 (63.2) | 68 (70.8) |  |
| None | 37 (36.6) | | 21 (42.0) | 16 (31.4) | 0.268 |  | 63 (30.0) | 35 (36.8) | 28 (29.2) | 0.2593 |
|  |  | |  |  |  |  |  |  |  |  |
| Reported hand washing of index case (times/day), mean (+/- SD), mean (+/- SD) | 2.9 (+/- 1.9) | | 3.1 (+/-2.4) | 2.8 (+/-1.3) | 0.403 |  | 3.4 (+/-2.0) | 3.9 (+/-2.6) | 2.8 (+/-1.5) | 0.0010 |
| Above median | 32 (32.7) | | 21 (42.0) | 11 (22.9) |  |  | 69 (37.9) | 49 (51.6) | 20 (23.0) |  |
| Less than or equal to median | 66 (67.3) | | 29 (58.0) | 37 (77.1) | 0.044 |  | 113 (62.1) | 46 (48.4) | 67 (77.0) | ≤0.0001 |
|  | | |  |  |  |  |  |  |  |  |
|  | |  |  |  |  |  |  |  |  |  |

Supplemental table 2a. Stratified analysis of hand washing versus control arm and surface contamination with influenza viruses, 2009-2010, Bangkok, Thailand

|  |  |  |  |  |  |  |  |  |  |  |
| --- | --- | --- | --- | --- | --- | --- | --- | --- | --- | --- |
|  |  | Proportion (%) of households with surface contamination by study arm | | |  |  |  |  |  |  |
|  |  |  |  |  |  | PRD | Unadjusted | p for effect | Adjusted PRD^b^ | Adjusted |
| Variable |  | Control |  | Hand washing |  | (%) | P^a^ | modification | % (95% CI) | P |
| Crude |  | 17/96 (17.7) |  | 7/95 (7.4) |  | 10.3% | 0.048 |  |  |  |
| Age (years) |  |  |  |  |  |  |  |  |  |  |
| ≤ 6 |  | 8/47 (17.0) |  | 6/49 (12.2) |  | 4.8% | 0.572 | 0.212 | 10.5 (1.2-19.7) | 0.030 |
| > 6 |  | 9/49 (18.4) |  | 1/46 (2.2) |  | 16.2% | 0.016 |  |  |  |
| Gender of Index Case |  |  |  |  |  |  |  |  |  |  |
| Female |  | 9/40 (22.5) |  | 4/44 (9.1) |  | 13.4% | 0.131 | 0.601 | 10.6 (1.4-19.9) | 0.028 |
| Male |  | 8/56 (14.3) |  | 3/51 (5.9) |  | 8.4% | 0.208 |  |  |  |
| Influenza |  |  |  |  |  |  |  |  |  |  |
| Seasonal (H3N2, H1N1, B) |  | 7/36 (19.4) |  | 4/40 (10.0) |  | 9.4% | 0.332 | 0.857 | 10.51 (1.3-19.8) | 0.029 |
| A(H1N1)2009pdm |  | 10/60 (16.7) |  | 3/55 (5.5) |  | 11.2% | 0.078 |  |  |  |
| Dew point in household |  |  |  |  |  |  |  |  |  |  |
| ≤ 24.13 (°C) |  | 15/50 (30.0) |  | 3/43 (7.0) |  | 23.0% | 0.007 | 0.001 | 9.5 (0.2-18.7) | 0.047 |
| > 24.13 (°C) |  | 2/46 (4.35) |  | 4/51 (7.8) |  | -3.5% | 0.680 |  |  |  |
| Secondary influenza infections in household | | |  |  |  |  |  |  |  |  |
| >1case |  | 9/28 (32.1) |  | 2/35 (5.7) |  | 26.4% | 0.008 | 0.032 | 11 (1.5-20.4) | 0.023 |
| None |  | 8/68 (11.8) |  | 5/60 (8.3) |  | 3.4% | 0.571 |  |  |  |
| NOTE. CI, confidence interval; PRD, prevalence risk difference | | | | | | | | | | |
| ^a^ Fisher's exact method |  |  |  |  |  |  |  |  |  |  |
| ^b^ Prevalence of positive surface contamination risk difference between control arm and hand washing arm, adjusted (using Mantel-Haenszel method) for corresponding variable. | | | | | | | | | | |

Supplemental table 2b. Stratified analysis of absolute humidity (high vs. low dew point) and household surface contamination with influenza viruses, 2009-2010, Bangkok, Thailand

|  | | | | | | | | | | |
| --- | --- | --- | --- | --- | --- | --- | --- | --- | --- | --- |
|  |  | Proportion (%) of households with surface contamination by absolute humidity (AH) | | |  |  |  |  |  |  |
|  |  |  |  |  |  | PRD | Unadjusted | p for effect | Adjusted PRD^b^ | Adjusted |
| Variable |  | Low AH |  | High AH |  | (%) | P^a^ | modification | % (95% CI) | P |
| Crude |  | 18/93 (19.4) |  | 6/97(6.2) |  | 13.2% | 0.008 |  |  |  |
| Age |  |  |  |  |  |  |  |  |  |  |
| ≤ 6 |  | 9/44 (20.5) |  | 5/51 (9.8) |  | 10.7% | 0.160 | 0.549 | 13.4 (3.5-22.2) | 0.006 |
| > 6 |  | 9/49 (18.4) |  | 1/46 (2.2) |  | 16.2% | 0.016 |  |  |  |
| Gender of Index Case |  |  |  |  |  |  |  |  |  |  |
| Female |  | 10/45 (22.2) |  | 3/39 (7.7) |  | 14.5% | 0.078 | 0.753 | 12.8 (3.5-22.9) | 0.008 |
| Male |  | 8/48 (16.3) |  | 3/58 (5.2) |  | 11.5% | 0.063 |  |  |  |
| Influenza |  |  |  |  |  |  |  |  |  |  |
| Seasonal (H3N2, H1N1,B) |  | 9/34 (26.5) |  | 2/41 (4.9) |  | 21.6% | 0.018 | 0.179 | 13.4 (4.0-22.9) | 0.006 |
| A(H1N1)2009pdm |  | 9/59 (15.3) |  | 4/56 (7.14) |  | 8.1% | 0.241 |  |  |  |
| Secondary influenza infections in household | | |  |  |  |  |  |  |  |  |
| >1case |  | 10/29 (34.5) |  | 1/34 (2.9) |  | 31.5% | 0.002 | 0.011 | 13.5 (3.9-23.0) | 0.0052 |
| None |  | 8/64 (12.5) |  | 5/63 (7.9) |  | 4.6% | 0.560 |  |  |  |
| NOTE. CI, confidence interval; PRD, prevalence risk difference | | | | | | | | | | |
| ^a^ Fisher's exact method |  |  |  |  |  |  |  |  |  |  |
| ^b^ Prevalence of positive surface contamination risk difference between low (below median) and high (above median) absolute humidity, adjusted (using Mantel-Haenszel method) for corresponding variable. | | | | | | | | | | |
